# Comparative Efficacy of Metformin and Combined Oral Contraceptives in the Management of Adolescent Polycystic Ovary Syndrome: A Systematic Review of Randomized Controlled Trials

**DOI:** 10.7759/cureus.83850

**Published:** 2025-05-10

**Authors:** Aqsa Mandvia, Rebecca George, Komal Kumari, Katee Kumari, Leen I Sabbagh, Amritveer Bhullar, Danyah Mohamed Ismail, Nikhil Deep Kolanu, Amir Ali

**Affiliations:** 1 Obstetrics and Gynaecology, Gloucester Royal Hospital, Gloucester, GBR; 2 Obstetrics and Gynaecology, Malankara Orthodox Syrian Church Medical College, Kochi, IND; 3 Internal Medicine, Akbar Niazi Teaching Hospital, Islamabad, PAK; 4 Gynecology and Obstetrics, Dubai Medical College for Girls, Dubai, ARE; 5 Internal Medicine, Dr. D. Y. Patil Medical College, Pimpri-Chinchwad, IND; 6 Internal Medicine, Indira Gandhi Medical College and Research Institute, Puducherry, IND; 7 Internal Medicine, China Medical Univesity, Shenyang, CHN; 8 Internal Medicine, Services Hospital Lahore, Lahore, PAK

**Keywords:** adolescents, insulin resistance, metformin, oral contraceptives, ovulation, polycystic ovary syndrome, randomized controlled trials, spiomet, systematic review

## Abstract

This systematic review explores the comparative efficacy of metformin, combined oral contraceptives (COCs), and their combination in the management of polycystic ovary syndrome (PCOS) among adolescent females. A comprehensive literature search was conducted across PubMed, Scopus, and Cochrane CENTRAL in accordance with the Preferred Reporting Items for Systematic Reviews and Meta-Analyses (PRISMA) guidelines, yielding 645 records, of which seven randomized controlled trials (RCTs) met the inclusion criteria. The included studies varied in design, population size, and outcome focus but consistently evaluated metabolic, hormonal, and reproductive endpoints. Results indicated that, while COCs were effective in reducing hyperandrogenic symptoms and regulating menstrual cycles, they were often associated with adverse metabolic effects, such as increased hepatic enzymes and insulin resistance. In contrast, insulin-sensitizing agents, particularly in combination regimens such as SPIOMET (spironolactone, pioglitazone, and metformin combination), showed superior outcomes in improving ovulation rates, insulin sensitivity, and body composition, with fewer metabolic side effects. Combination therapy with metformin and COCs also demonstrated a balanced improvement in both reproductive and metabolic profiles. The overall quality of included studies was rated as having some concerns due to variability in study design and reporting. Despite these limitations, the evidence supports a more individualized and metabolically focused approach to managing adolescent PCOS, emphasizing the benefits of early intervention with insulin-sensitizing therapies.

## Introduction and background

Polycystic ovary syndrome (PCOS) is a prevalent endocrine disorder affecting adolescent females worldwide, characterized by a constellation of clinical features, including hyperandrogenism, oligo-anovulation, and polycystic ovarian morphology [1,2]. The pathophysiology of PCOS in adolescents is complex and often overlaps with normal pubertal changes, making diagnosis and management particularly challenging [3]. In addition to its reproductive implications, PCOS is associated with significant metabolic disturbances, such as insulin resistance, obesity, dyslipidemia, and an increased risk for type 2 diabetes mellitus. Early intervention is essential not only to address menstrual irregularities and dermatological manifestations such as acne and hirsutism but also to mitigate long-term cardiovascular and metabolic risks [4].

Current therapeutic strategies for managing PCOS in adolescents include hormonal agents, such as combined oral contraceptive pills (COCs), which are often the first-line treatment for regulating menstrual cycles and reducing androgen-related symptoms [5]. On the other hand, insulin-sensitizing agents, such as metformin, have gained increasing attention due to their favorable impact on metabolic parameters and potential to improve ovulatory function [6]. In some cases, a combination of metformin and COCs is employed to address both reproductive and metabolic components of the syndrome. However, the optimal treatment approach remains debated, particularly in the adolescent population, where long-term safety, tolerability, and effectiveness must be carefully weighed. While numerous clinical trials have evaluated these therapies individually or in combination, a systematic synthesis of evidence focusing specifically on adolescents is lacking. Therefore, this systematic review aims to compare the efficacy of metformin, combined oral contraceptives, and their combination in managing adolescent PCOS, with an emphasis on both metabolic and reproductive outcomes. This systematic review is guided by the PICO framework [7], focusing on adolescents with PCOS and evaluating the comparative effectiveness of metformin, combined oral contraceptives, and their combination on both metabolic and reproductive outcomes.

## Review

Materials and methods

Search Strategy

The search strategy for this systematic review was designed in accordance with the Cochrane Handbook for Systematic Reviews of Interventions to ensure methodological rigor in study identification and selection. Reporting of the review followed the Preferred Reporting Items for Systematic Reviews and Meta-Analyses (PRISMA) 2020 checklist [8]. The review protocol was not prospectively registered, which we acknowledge as a limitation and plan to address in future studies to enhance transparency and reproducibility.

A comprehensive search was conducted across PubMed, Scopus, and Cochrane CENTRAL, covering literature published from January 2014 to January 2024. This 10-year window was selected to reflect contemporary treatment trends and recent advances in adolescent PCOS management. No filters were applied during the search process aside from language (English) and human studies to maintain sensitivity and relevance. Search terms included both Medical Subject Headings (MeSH) and free-text keywords such as “Polycystic Ovary Syndrome,” “Adolescents,” “Metformin,” “Oral Contraceptives,” “Combined Therapy,” and “Randomized Controlled Trial.”

All retrieved citations were imported into a reference manager, and duplicates were removed. Two independent reviewers screened titles and abstracts, followed by full-text assessment against predefined inclusion criteria. Discrepancies were resolved through discussion or by consulting a third reviewer when necessary. Only randomized controlled trials (RCTs) involving adolescent females with PCOS and directly comparing metformin, COCs, or their combination were included. Data extraction and quality assessment were also conducted independently by two reviewers.

Eligibility Criteria

The review was structured using the PICO (population, intervention, comparison, outcome) framework. The Population consisted of adolescent females diagnosed with polycystic ovary syndrome. The interventions assessed were metformin monotherapy, COCs alone, or a combination of both. The comparisons were drawn among these therapeutic strategies to evaluate their relative effectiveness. The outcomes included menstrual regularity, ovulation rates, clinical hyperandrogenism (e.g., acne, hirsutism), insulin sensitivity, body mass index, and quality of life metrics.

The eligibility criteria for this systematic review were carefully defined to ensure alignment with the research objective-comparing the efficacy of metformin, COCs, and their combination in managing PCOS within adolescent populations. Only RCTs published in English within the last 10 years were considered. Studies were included if they involved female participants diagnosed with PCOS, primarily in the adolescent age group (≤19 years). However, studies including participants aged up to 22 years were also considered, provided that the mean age remained within the late adolescent to early adulthood range and the reported outcomes were directly applicable to adolescent PCOS management. This inclusion reflects the transitional age group where PCOS often first manifests and is managed similarly in clinical practice. Studies were required to evaluate at least one relevant clinical, hormonal, or metabolic outcome (e.g., ovulation rate, insulin resistance, BMI, free testosterone levels, or quality of life) and to clearly describe the intervention as metformin, COCs, or their combination.

Exclusion criteria included non-randomized studies, reviews, case reports, editorials, and trials focusing exclusively on adult women without relevance to adolescent physiology or management. Additionally, studies lacking sufficient data on treatment outcomes or those using non-standardized or unclear diagnostic criteria for PCOS were excluded. Studies involving additional interventions such as laser therapy or lifestyle modification were excluded unless they allowed for clear differentiation of pharmacological effects. This strict yet balanced eligibility framework ensured the inclusion of high-quality evidence while maintaining clinical applicability to the adolescent PCOS population.

Data Extraction

Data extraction was performed systematically using a predefined template designed to capture all relevant study characteristics and outcomes in accordance with PRISMA guidelines. For each included RCT, information was collected on the study design, population demographics (including sample size and PCOS diagnostic criteria), intervention and comparison groups, treatment duration, and outcomes assessed. Specific outcomes of interest included clinical (e.g., menstrual regularity, ovulation rate, hirsutism), metabolic (e.g., BMI, insulin resistance, hepatic markers), hormonal (e.g., free testosterone, follistatin), and patient-reported outcomes such as quality of life. Key findings and limitations reported by the study authors were also extracted to inform qualitative synthesis and critical appraisal. Data were cross-verified to ensure consistency, and any discrepancies were resolved through discussion among the reviewers to ensure accuracy and minimize extraction bias.

Data Analysis and Synthesis

Data analysis and synthesis were conducted through a qualitative narrative approach due to heterogeneity in study populations, intervention protocols, outcome measures, and follow-up durations across the included trials. The findings from each study were systematically compared and grouped based on intervention type - metformin alone, COCs alone, or combination therapy - and key outcomes such as ovulation rates, metabolic markers, hormonal levels, and quality of life. Given the variation in outcome reporting formats and the limited number of adolescent-focused RCTs, a meta-analysis was not feasible. Instead, thematic synthesis allowed for the identification of consistent patterns, clinical trends, and treatment effects across studies. As part of the synthesis process, the methodological quality of the included trials was assessed using the Cochrane Risk of Bias 2 (RoB 2) tool, and studies were interpreted in light of their respective risk of bias to support a balanced and evidence-based comparison of findings.

Results

Study Selection Process

**Figure 1:**
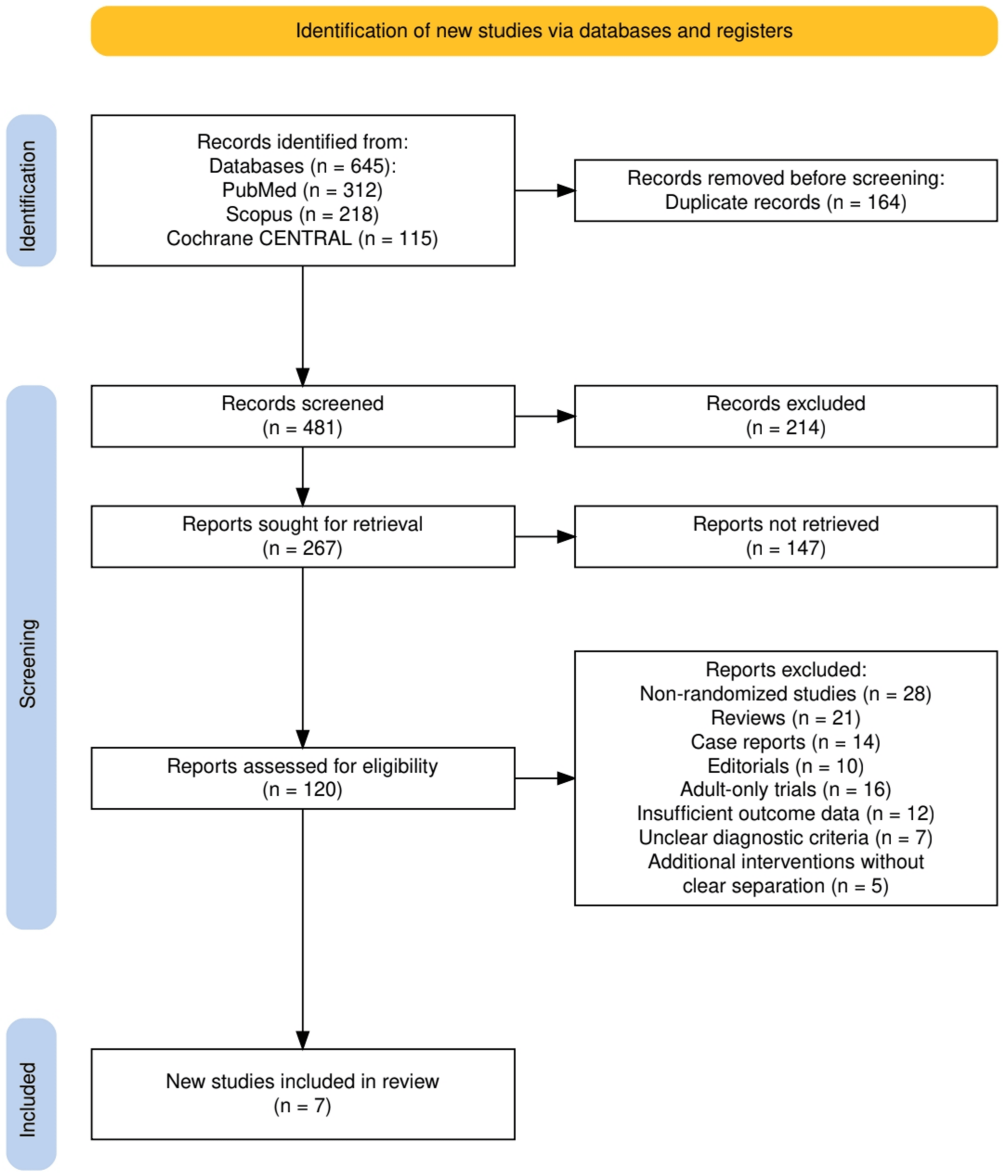
The PRISMA flowchart represents the study selection process. PRISMA: Preferred Reporting Items for Systematic Reviews and Meta-Analyses

The study selection process is summarized in Figure 1, which outlines the flow of literature through each stage of the review following PRISMA 2020 guidelines. A total of 645 records were initially identified across three databases: PubMed (n = 312), Scopus (n = 218), and Cochrane CENTRAL (n = 115). After removing 164 duplicate records, 481 titles and abstracts were screened, resulting in the exclusion of 214 irrelevant articles. Of the 267 reports sought for retrieval, 147 could not be obtained, leaving 120 full-text articles assessed for eligibility. Among these, 113 were excluded for reasons such as non-randomized study design, irrelevant population (adult-only), insufficient outcome data, or inclusion of additional interventions without clear separation. Ultimately, seven RCTs met all inclusion criteria and were included in the final qualitative synthesis.

Characteristics of the Selected Studies

The characteristics of the studies included in this review are summarized in Table 1, which provides a detailed overview of study design, population demographics, intervention and comparison groups, treatment duration, measured outcomes, key findings, and limitations. All seven studies were RCTs, with three explicitly focused on adolescent girls with PCOS and the remaining four including populations that likely encompassed older adolescents and young adults. Intervention strategies varied across studies, encompassing metformin monotherapy, COCs, and combination regimens such as SPIOMET or metformin + OCP. Treatment durations ranged from six to 12 months, with some studies extending follow-up into post-treatment periods. The outcomes measured included a broad spectrum of clinical, hormonal, metabolic, and patient-reported variables. Collectively, the studies demonstrated variable efficacy across interventions, with insulin-sensitizing agents - particularly in combination with anti-androgenic or hormonal therapy - showing promising results in improving metabolic profiles and reproductive function. However, heterogeneity in population age, diagnostic criteria, and endpoints, along with small sample sizes in several trials, highlighted key methodological limitations.

**Table 1:** Summary of randomized controlled trials comparing metformin, COCs, and combination therapies in adolescent PCOS. ALT: Alanine Aminotransferase; AST: Aspartate Aminotransferase; BMI: Body mass index; DBI: Diazepam-binding inhibitor; DXA: Dual-energy X-ray absorptiometry; EE-CA: Ethinylestradiol–cyproterone acetate; EE-LNG: Ethinylestradiol–levonorgestrel; FGF21: Fibroblast growth factor 21; FG Score: Ferriman-Gallwey score; FT: Free testosterone; GGT: Gamma-glutamyl transferase; HRQoL: Health-related quality of life; IL-6: Interleukin-6; METRNL: Meteorin-like potein; MCP-1: Monocyte chemoattractant protein-1; OC: Oral contraceptive; OCP: Oral contraceptive pill; PCOS: Polycystic vary syndrome; RCT: Randomized controlled trial; SPIOMET: Spironolactone, pioglitazone, and metformin combination

Study (Author, Year)	Study Design	Population (Age, Sample Size, PCOS Criteria)	Intervention Group(s)	Comparison Group(s)	Duration of Treatment	Outcomes Measured	Key Findings	Limitations
Garcia-Beltran et al., 2024 [9]	Randomized Pilot Study	Adolescent girls with PCOS and without obesity, N=54 (OC=26, Spiomet=28); healthy controls N=17; diagnostic criteria not stated	OC, Spiomet (spironolactone + pioglitazone + metformin)	OC vs Spiomet; controls for reference	6 months (organokines), 12 months (liver enzymes)	Liver enzymes (ALT, AST, GGT), organokines (FGF21, DBI, METRNL), metabolic markers	OC raised ALT & GGT; spiomet did not. DBI lower in OC vs spiomet and controls. FGF21 elevated in PCOS. METRNL correlated with liver enzymes in OC group.	Small sample; organokines were post hoc; lacks reproductive/clinical outcomes
Díaz et al., 2023 [10]	RCT	Adolescent girls with PCOS, N=72; mean age 16, BMI ~23 kg/m²; diagnostic criteria not stated	SPIOMET, PioFluMet, EE-CA, EE-LNG	Healthy controls (n=28) and comparison across groups	6 months	Serum follistatin, insulin resistance, liver fat, metabolic markers	OCs increased follistatin (6.8x in EE-CA, 2.5x in EE-LNG); SPIOMET and PioFluMet did not. Follistatin linked to insulin resistance and liver fat.	Biomarker focus; no clinical symptom outcomes (e.g. menses, hirsutism)
Ibáñez et al., 2017 [11]	Randomized, Open-Label Pilot Study	Adolescent girls with PCOS (NIH), N=36; mean age 16, BMI ~23.5 kg/m²; 94% completion	SPIOMET (spironolactone + pioglitazone + metformin)	OC (ethinylestradiol + levonorgestrel)	12 months treatment + 12 months off-treatment	Ovulation (diary + salivary progesterone), hepatic/visceral fat, insulin, androgens	SPIOMET had 2.5× higher ovulation rate and 6× more normovulation than OC. Visceral fat and insulin improved only with SPIOMET. OC reduced androgens faster but rebounded.	Small size; open-label; not powered for long-term outcomes
Altinok et al., 2018 [12]	RCT	Women with PCOS, N=90; age unspecified; likely includes young adults; PCOS criteria not given	Metformin, OCP, Metformin + OCP	Each other	12 months	HRQoL (PCOS-VAS, SF-36), BMI, FG score	HRQoL improved in all groups. Facial hair scores improved more with OCP and combo vs metformin. M and M+OCP reduced weight. FG score improved with combo.	Not adolescent-specific; subjective endpoints; lacks ovulatory/hormonal measures
Al-Zubeidi & Klein, 2015 [13]	RCT	Adolescent girls with PCOS, N=22; diagnostic criteria not specified	Metformin	OCP	6 months + 3 months post-treatment	BMI, Free Testosterone (FT), Insulin resistance, Menses	BMI dropped in both groups; FT decreased significantly only in OCP. Menstrual frequency improved with OCP. BMI/FT improvements lasted post-treatment.	Small sample; limited duration; minimal metabolic/hormonal detail
Glintborg et al., 2014 (JCEM) [14]	RCT	Women with PCOS, N=90 randomized (65 completed); age not specified	Metformin, Metformin + OCP	OCP	12 months	Weight, regional fat mass (DXA), Free Testosterone (FT)	M and M+OCP led to weight loss and better fat profile than OCP. FT decreased more with M+OCP and OCP.	Not adolescent-specific; dropout rate; diagnostic detail not in abstract
Glintborg et al., 2014 (JEI) [15]	RCT	Same population as above; N=90 randomized (65 completed)	Metformin, Metformin + OCP	OCP	12 months	Adiponectin, IL-6, MCP-1, trunk fat (DXA)	M and M+OCP improved fat mass vs OCP. Inflammatory markers unchanged. Trunk fat changes linked to IL-6/MCP-1 only in M+OCP group.	Biomarker-focused; no reproductive outcomes; not adolescent-specific

Quality Assessment

The quality assessment of the included studies was conducted using the RoB 2.0 tool [16], and the results are summarized in Table 2. Overall, all seven RCTs were rated as having "some concerns" regarding risk of bias, with none classified as either low or high risk overall. The most common issues across studies included limited clarity regarding the randomization process and allocation concealment, as well as occasional lack of transparency in reporting. One study was open-label, introducing a higher risk of performance bias due to deviations from intended interventions. Minor concerns regarding missing outcome data were present in studies with small sample sizes or incomplete follow-up reporting. Measurement of outcomes was generally well-executed and consistent, although a few studies relying heavily on subjective endpoints, such as quality of life assessments, raised concerns about selective reporting. Despite these limitations, the overall methodological quality of the included trials was acceptable, supporting their inclusion in the final synthesis and the reliability of the conclusions drawn from their findings.

**Table 2:** Risk of bias assessment of the included studies using the Cochrane RoB 2.0 tool.

Study (Author, Year)	Randomization Process	Deviations from Intended Interventions	Missing Outcome Data	Measurement of Outcome	Selective Reporting	Overall Risk of Bias	Notes
Garcia-Beltran et al., 2024 [9]	Some Concerns	Low Risk	Low Risk	Low Risk	Some Concerns	Some Concerns	Small sample; organokines were post hoc; details of allocation unclear
Díaz et al., 2023 [10]	Some Concerns	Low Risk	Low Risk	Low Risk	Low Risk	Some Concerns	Well-conducted but lacks detail on allocation sequence concealment
Ibáñez et al., 2017 [11]	Some Concerns	High Risk (open-label)	Low Risk	Low Risk	Low Risk	Some Concerns	Open-label design increases performance bias
Altinok et al., 2018 [12]	Some Concerns	Low Risk	Low Risk	Low Risk	Some Concerns	Some Concerns	Subjective outcomes; unclear allocation method; not adolescent-specific
Al-Zubeidi & Klein, 2015 [13]	Some Concerns	Low Risk	Some Concerns	Low Risk	Low Risk	Some Concerns	Small sample; follow-up data available but reporting was brief
Glintborg et al., 2014 (JCEM) [14]	Low Risk	Low Risk	Some Concerns	Low Risk	Low Risk	Some Concerns	Dropouts were present; ITT analysis not confirmed
Glintborg et al., 2014 (JEI) [15]	Low Risk	Low Risk	Some Concerns	Low Risk	Low Risk	Some Concerns	Similar cohort as JCEM study; secondary biomarker focus

Discussion

The studies included in this systematic review collectively examined the comparative efficacy of metformin, COCs, and their combinations in managing PCOS in adolescent and young female populations. Across several trials, SPIOMET (a combination of spironolactone, pioglitazone, and metformin) consistently demonstrated favorable metabolic and reproductive outcomes. In particular, Ibáñez et al. [11] found that SPIOMET significantly improved ovulation rates and normalized visceral fat and insulin levels compared to COCs, which, although effective in reducing androgen levels, showed post-treatment rebound. Similarly, García-Beltran et al. [9] and Díaz et al. [10] reported that SPIOMET and related insulin-sensitizing combinations did not elevate hepatic enzymes or follistatin levels, in contrast to COCs, which were associated with liver stress and markers of insulin resistance.

Other trials directly comparing metformin, COCs, and their combination revealed a consistent trend: metformin, either alone or combined with COCs, was superior in improving body weight and fat distribution, while COCs alone were more effective in lowering free testosterone levels and improving menstrual frequency. Notably, Altinok et al. [12] and Glintborg et al. [14] reported improvements in body composition and quality of life across all groups, with greater reductions in weight and FG scores observed in combination therapy groups. Al-Zubeidi et al. [13] specifically highlighted the effectiveness of COCs in reducing androgen levels and improving menstrual regularity in adolescents, although metformin had sustained benefits post-treatment. Overall, the review indicates that, while COCs effectively manage hyperandrogenism, insulin-sensitizing agents - particularly in combination - offer superior metabolic and long-term reproductive benefits in adolescents with PCOS [17].

The findings of this systematic review align with existing literature that highlights the limitations of monotherapy with COCs in managing the multifactorial nature of polycystic ovary syndrome, particularly in adolescents. Previous studies have consistently demonstrated that while COCs are effective in regulating menstrual cycles and reducing clinical signs of hyperandrogenism, their metabolic profile remains suboptimal, often associated with weight gain, elevated liver enzymes, and adverse effects on insulin sensitivity [18,19]. The included studies, particularly those by García-Beltran et al. [9] and Díaz et al. [10], reinforce these concerns, showing elevated markers of hepatic stress and insulin resistance with COC use. These outcomes are consistent with the broader body of endocrinology literature, which cautions against relying solely on hormonal contraceptives for adolescents with PCOS [20], especially those with underlying metabolic dysfunction.

In contrast, the metabolic benefits of insulin-sensitizing agents such as metformin and combination therapies such as SPIOMET have been increasingly emphasized in contemporary research. The improvements in ovulation rate, visceral adiposity, and insulin sensitivity observed in the SPIOMET arms of the included trials mirror findings from earlier non-randomized trials and cohort studies that advocate for early metabolic intervention in PCOS management [21]. Studies such as those by Ibáñez et al. [11] have also contributed to the growing body of evidence supporting the role of insulin sensitizers in not only mitigating metabolic risk factors but also improving long-term reproductive outcomes. Furthermore, the additive effect of metformin when combined with COCs, as seen in the studies by Glintborg et al. [14], supports previous observations that combination therapy may offer a balanced approach by addressing both hormonal and metabolic aspects of PCOS more effectively than either agent alone. These findings collectively suggest a paradigm shift in clinical recommendations, favoring individualized, pathophysiology-based treatment strategies over traditional hormone-centric approaches.

The results of this systematic review suggest that, while combined oral contraceptives remain effective for addressing hyperandrogenic symptoms, such as hirsutism and menstrual irregularities, they may not adequately target the underlying metabolic disturbances in adolescent PCOS [22]. In contrast, insulin-sensitizing therapies, particularly SPIOMET and metformin-based regimens, appear to offer a more comprehensive benefit by improving ovulation rates, reducing hepatic and visceral adiposity, enhancing insulin sensitivity, and minimizing adverse hepatic effects [23]. The observed superiority of SPIOMET in achieving normovulation and favorable metabolic outcomes underscores the importance of early interventions targeting the root metabolic drivers of PCOS rather than solely its hormonal manifestations. Furthermore, the additive benefit of combining metformin with COCs, as seen in several trials, supports a dual-approach strategy that can simultaneously address both reproductive and metabolic dimensions of the syndrome. These findings emphasize the need for a more individualized, multifaceted treatment approach, especially in adolescent populations at risk of long-term cardiometabolic complications.

The findings of this review carry important clinical and public health implications, particularly in guiding the early management of PCOS in adolescent populations. Given the rising prevalence of PCOS and its association with long-term risks, such as type 2 diabetes, cardiovascular disease, and infertility, timely and targeted intervention is crucial. The evidence supports a shift away from a one-size-fits-all reliance on combined oral contraceptives toward more individualized treatment plans that incorporate insulin-sensitizing agents such as metformin, either alone or in combination with hormonal therapies [24]. Clinically, this approach can lead to more sustainable improvements in both metabolic health and reproductive function. From a public health perspective, early metabolic intervention in adolescents with PCOS has the potential to reduce future healthcare burdens associated with obesity-related comorbidities, improve quality of life, and support healthier reproductive trajectories in young women [25].

A key strength of this systematic review is its exclusive focus on randomized controlled trials, which enhances the overall reliability of the synthesized evidence. By examining studies that include adolescent populations and comparing metformin, COCs, and combination therapies, the review provides a more nuanced understanding of treatment strategies in a group often underrepresented in PCOS research. The inclusion of diverse outcome domains - reproductive, metabolic, hormonal, and quality-of-life further strengthens the comprehensiveness of the analysis.

However, several limitations should be acknowledged. Some included studies had small sample sizes, lacked detailed reporting of randomization methods or PCOS diagnostic criteria, and, in a few cases, extended slightly beyond the strict adolescent age group, which may impact generalizability. The heterogeneity in study design, treatment protocols, and outcome measures limited the feasibility of conducting a meta-analysis and may have introduced variability in interpreting treatment effects. Additionally, performance and selection biases cannot be ruled out due to open-label designs and incomplete blinding in some trials. The exclusion of studies due to inaccessible full texts, despite multiple retrieval efforts, may also have introduced a minor risk of selection bias.

Future research should prioritize large-scale, multicenter RCTs focused exclusively on adolescents with clearly defined diagnostic criteria for PCOS. Studies should aim to assess both short- and long-term outcomes, including ovulatory function, fertility potential, metabolic risk trajectories, and psychosocial well-being. There is also a need for standardized outcome reporting and harmonized follow-up durations to allow for cross-study comparison and evidence synthesis. Additionally, head-to-head trials comparing conventional therapies with emerging approaches - such as novel insulin-sensitizing agents, lifestyle modification, and nutraceuticals - will be essential to developing individualized, pathophysiology-driven treatment strategies for adolescent PCOS.

## Conclusions

This systematic review highlights the nuanced therapeutic landscape of managing PCOS in adolescents, emphasizing that, while combined oral contraceptives are effective for addressing hyperandrogenic symptoms, insulin-sensitizing agents - particularly when used in combination - offer superior benefits in terms of metabolic health, ovulatory restoration, and long-term risk reduction. The evidence supports a shift toward more personalized, pathophysiology-based treatment approaches that go beyond symptomatic control to address the underlying insulin resistance and adiposity often present in adolescent PCOS. Clinicians should consider integrating metformin-based regimens early in the management plan, especially for patients with metabolic abnormalities, to optimize both short-term symptom relief and long-term health outcomes.
